# U.S. Science Politics are Top Headlines in Both Journals

**DOI:** 10.1100/tsw.2001.69

**Published:** 2001-06-29

**Authors:** Shauna M. Haley

## Abstract

Nature and Science open the news week with an introduction to U.S. President Bush's choice for the nation's science advisor post as their top subject. Both journals also report in their second slots on the same administration's difficulties developing national stem cell and cloning policies.

John H. Marburger III, a physicist, was officially announced as the Bush administration's choice for the United States' top presidential science advisor post earlier this week. If confirmed by the Senate in the fall, Marburger will become the director of the Office of Science and Technology Policy. The Ivy league grad currently runs the Brookhaven National Laboratory, where he arrived to quell public fury over a radioactive leak, reports *Nature*. *Science* observes that Marburger was also the leader of the Universities Research Association in the heated political battles over the now-defunct Superconducting Super Collider project. Peers interviewed by both journals seem to support Marburger, characterizing him as a diversified, politically savvy scientist who understands the complex relationship between science and government. Both journals report speculation about whether the director-in-waiting will be able to mingle well with the closed Washington crowd and have access to the president. Curiously, although stem cell decisions and climate change are now priority for Bush, only *Science* notes Marburger's diplomatic comments on those issues.

Reports of intensifying debates over whether or not the U.S. government should fund human embryonic stem cell research carry both second positions in *Nature* and *Science*. *Nature*'s briefer story centers on contention in the scientific community about recent research implying adult stem cells are as malleable as embryonic stem cells. Current projects have not been able to replicate some experiments, says *Nature*. Two major forces in Bush's own conservative political party appear to support at least the research on embryonic stem cells – one, a vocal antiabortion supporter, wrote a letter urging the president to allow government funding, reports *Science*. Developmental biologist, Margaret Goodell, whose 1999 paper on muscle stem cell differentiation is often cited as evidence for adult stem cell potential, told *Science* that in a newly submitted research report, she found that muscle actually contains more than one kind of fated cell. Hence, her original findings can no longer be touted as evidence for the equivalent-potential argument being used by politicians and other scientists to oppose federal funding of embryonic stem cell research. While the therapeutic possibilities for stem cells seems clear, politicians, not scientists, will determine the direction of research in the U.S. with the bang of the buck.
